# Exploring the Impact of Extracorporeal Membrane Oxygenation on the Endothelium: A Systematic Review

**DOI:** 10.3390/ijms251910680

**Published:** 2024-10-03

**Authors:** Yakun Li, Carolien Volleman, Dionne P. C. Dubelaar, Alexander P. J. Vlaar, Charissa E. van den Brom

**Affiliations:** 1 Department of Intensive Care Medicine, Amsterdam UMC, University of Amsterdam, 1105 AZ Amsterdam, The Netherlands; yakunli96@gmail.com (Y.L.); c.volleman@amsterdamumc.nl (C.V.); d.p.c.dubelaar@amsterdamumc.nl (D.P.C.D.); a.p.vlaar@amsterdamumc.nl (A.P.J.V.); 2 Laboratory of Experimental Intensive Care and Anesthesiology (LEICA), Amsterdam UMC, University of Amsterdam, 1105 AZ Amsterdam, The Netherlands; 3 Department of Anesthesiology, Amsterdam UMC, VU University Amsterdam, 1081 HV Amsterdam, The Netherlands

**Keywords:** endothelium, endothelial activation, endothelial damage, permeability, glycocalyx, microvascular leakage, edema, extracorporeal membrane oxygenation, extracorporeal circulation

## Abstract

Extracorporeal membrane oxygenation (ECMO) is a life-saving intervention for patients with circulatory and/or pulmonary failure; however, the rate of complications remains high. ECMO induces systemic inflammation, which may activate and damage the endothelium, thereby causing edema and organ dysfunction. Advancing our understanding in this area is crucial for improving patient outcomes during ECMO. The goal of this review is to summarize the current evidence of the effects of ECMO on endothelial activation and damage in both animals and patients. PubMed and Embase databases were systematically searched for both clinical and animal studies including ECMO support. The outcome parameters were markers of endothelial activation and damage or (in)direct measurements of endothelial permeability, fluid leakage and edema. In total, 26 studies (patient *n* = 16, animal *n* = 10) fulfilled all eligibility criteria, and used VA-ECMO (*n* = 13) or VV-ECMO (*n* = 6), or remained undefined (*n* = 7). The most frequently studied endothelial activation markers were adhesion molecules (ICAM-1) and selectins (E- and P-selectin). The levels of endothelial activation markers were comparable to or higher than in healthy controls. Compared to pre-ECMO or non-ECMO, the majority of studies showed stable or decreased levels. Angiopoietin-2, von Willebrand Factor and extracellular vesicles were the most widely studied circulating markers of endothelial damage. More than half of the included studies showed increased levels when compared to normal ranges, and pre-ECMO or non-ECMO values. In healthy animals, ECMO itself leads to vascular leakage and edema. The effect of ECMO support in critically ill animals showed contradicting results. ECMO support (further) induces endothelial damage, but endothelial activation does not, in the critically ill. Further research is necessary to conclude on the effect of the underlying comorbidity and type of ECMO support applied on endothelial dysfunction.

## 1. Introduction

Extracorporeal membrane oxygenation (ECMO) is a life-saving intervention for patients with circulatory and/or pulmonary failure unresponsive to conventional treatments. Over the last decade, the use of ECMO has doubled, and a total of 18,159 ECMO runs were performed worldwide in 2022 [1]. Despite technological advances and the promising role of ECMO, the rate of complications remains high, and only 54% of ECMO patients survived to hospital discharge or transfer in 2022 [1]. This highlights the critical need for an increased recognition and understanding of the potential negative effects of ECMO.

Among the many complications that can result from ECMO, the effect on the microcirculation as well as the endothelium remains a black box. ECMO is associated with a systemic inflammatory response due to, amongst others, blood-material interaction [2,3]. This inflammatory response can activate the endothelium [3], and as a consequence, endothelial permeability increases. This can result in fluid leakage to the interstitium, causing tissue edema, disturbances in microcirculatory perfusion and organ dysfunction. Moreover, to enable sufficient blood flow for the ECMO system to run, fluid resuscitation is often necessary. This fluid resuscitation can aggravate organ injury, presumably by increasing fluid leakage and tissue edema [4]. However, even though it is known from patients on cardiopulmonary bypass (CPB) that endothelial dysfunction is associated with organ dysfunction [5], this issue in patients on ECMO support is underappreciated.

The integrity of the endothelial monolayer is essential for adequate tissue perfusion and oxygenation, both fundamental goals of ECMO support. Microvascular dysfunction can result in tissue hypoxia and subsequent organ failure, undermining the therapeutic objectives of ECMO [6]. A recent scoping review highlighted endothelial dysfunction in adults on veno-arterial extracorporeal membrane oxygenation (VA-ECMO) as a significant complication, with experts from various fields identifying it as a top research priority [7]. Preliminary evidence of endothelial activation was summarized from patients on VA-ECMO support [7]; however, additional insights should also be obtained from patients on veno-venous (VV) ECMO. Besides patient data, animal studies should be taken into account to differentiate between the effects of extracorporeal circulation and the underlying disease to better elucidate the impact of solely ECMO on endothelial function.

In this review, we therefore aimed to summarize the current evidence on endothelial activation and dysfunction during VA- and VV-ECMO in both animals and patients, and identify areas of interest for future research.

## 2. Methods

### 2.1. Protocol and Registration

The protocol of this review was registered in the PROSPERO international prospective register of systematic reviews under the registration number CRD42023459453. This review was performed and reported according to the Preferred Reporting Items for Systematic Reviews and Meta-Analyses (PRISMA) guidelines [8].

### 2.2. Eligibility Criteria

This review included all patient and animal studies with any type or duration of ECMO support. Study protocols with any possible reasons for ECMO initiation and subjects of all ages were eligible for inclusion. The comparisons in this review include the following: (1) ECMO-supported changes overtime vs. baseline; (2) critically ill subjects with ECMO support vs. those without ECMO support; (3) ECMO-supported subjects (healthy or ill) vs. healthy controls; and (4) ECMO-supported patients with poor outcomes vs. those with good outcomes. Outcome parameters were markers for endothelial activation and damage or (in)direct measurements of endothelial permeability, fluid leakage, edema or glycocalyx degradation.

### 2.3. Search Strategy

In February 2023, PubMed and EMBASE were systematically searched for eligible studies in collaboration with a medical information specialist, and the search was re-run in April 2024. The full search strategy was based on a combination of the following search components: “extracorporeal membrane oxygenation”, “endothelial”, “leakage”, “edema”, “permeability” or “glycocalyx”. The search strategies are shown in Supplementary File S1. Non-English articles, reviews, meeting abstracts, conference reports, letters or editorials were excluded. 

### 2.4. Study Selection

The initial screening was based on the title and abstract retrieved using the search strategy, and was performed independently by two reviewers (Y.L. and C.V.). Duplicates were removed and screening results were organized. Full texts of potentially eligible studies were obtained and eligible studies were identified by two reviewers after reading full texts (Y.L. and C.V.). Any disagreements were resolved by discussion, and if necessary, discussed with a third reviewer (C.E.v.d.B.). The reference lists of included studies were screened for additional eligible studies not retrieved by our search. 

### 2.5. Data Extraction

Data extraction was performed by one reviewer (Y.L.) and confirmed by another (D.D.). Data from the included articles were extracted and collected using a data extraction form. For animal studies, study characteristics, animals (species, sex, size, and housing), group size and protocol specifications (injury type, ECMO type, connection, duration, flow rate, priming volume, anticoagulation, ventilation, and anesthesia methods) were extracted. For patient studies, the following information was extracted: study design, inclusion and exclusion criteria of the individual study, patient demographics, and characteristics of the ECMO run, including type and duration. Moreover, details regarding outcomes (technique, organ, time point and comparison) were collected.

### 2.6. Quality Assessment

The quality of studies and risk of bias were assessed using the NIH quality assessment tool for clinical studies [9]. The NIH quality assessment tool assesses selection, performance, detection, attrition, and reporting biases through a set list of yes or no questions. For preclinical studies, the methodological quality was assessed based on the Systematic Review Centre for Laboratory animal Experimentation (SYRCLE) risk of bias tool assessing selection, performance, detection, attrition, and reporting bias [10]. Quality assessment was performed independently by two reviewers (Y.L. and C.V.). Any discrepancies were resolved through discussion among them.

### 2.7. Study Measures and Analysis

The current review aimed to give an overview of the effect of ECMO on endothelial activation and damage. The outcome parameters included measurements of markers of endothelial activation and dysfunction, and (in)direct measurements of endothelial permeability, fluid leakage and edema. Due to the heterogeneity of the included studies, a meta-analysis was considered as unfeasible. Instead, a narrative synthesis of the results was conducted. 

## 3. Results

### 3.1. Search Results

**Figure 1:**
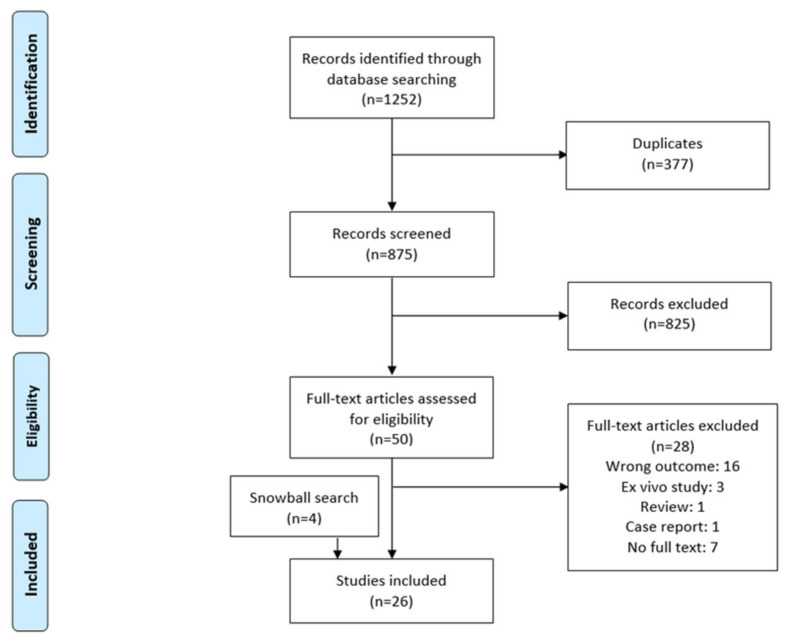
PRISMA diagram representing the flowchart of study selection. PRISMA, Preferred Reporting Items for Systematic Reviews.

A total of 1252 articles were identified through the search of PubMed and EMBASE. After the deletion of duplicates (*n* = 377), 875 records were screened for eligibility. The remaining 50 full texts were examined, and eventually 26 studies met the selection criteria. Sixteen patient studies and ten animal studies were included. Figure 1 presents the flow of evidence as a PRISMA diagram. Included articles were published between 2000 and 2023, and studies were performed in 10 different countries.

### 3.2. Study Characteristics

Of the sixteen included articles investigating endothelial function in patients on ECMO support (Supplementary File S2), thirteen studies were prospective trials and three studies were performed retrospectively. The number of patients on ECMO support varied between 10 and 132 per study. Nine of the studies were carried out in adults and seven were performed in patients under the age of 18. Five studies focused on VA-ECMO [11,12,13,14,15] and four studies focused on VV-ECMO [16,17,18,19], whereas seven studies did not differentiate between ECMO types [20,21,22,23,24,25,26].

Likewise, an overview of animal characteristics is presented in Supplementary File S3. Studies were performed in four different species. The majority of studies were performed in rats (*n* = 7) [27,28,29,30,31,32,33], one study used pigs [34], one study used dogs [35] and one study used rabbits [36]. Group size varied between 4 and 10 animals per group. The majority of the studies used male animals (80%), one study used female animals [30] and one study did not report sex [35]. Details of ECMO protocols used in these studies are summarized in Supplementary File S3. Most studies used VA-ECMO (*n* = 8), whereas two studies used VV-ECMO [27,28] with various initiation reasons. The flow rates of the pump ranged from 40 to 150 mL/kg/min in rats, 50 mL/kg/min in pigs, 130 mL/kg/min in dogs and less than 50 mL/kg/min in rabbits. Overall, the time of ECMO support ranged from 30 min to 6 h, with two exceptions in which the duration of ECMO assistance was not mentioned [30], or it was mentioned that animals were weaned from ECMO after return of spontaneous circulation (ROSC) [34].

### 3.3. Risk of Bias

The risk of bias assessment per study is provided in Supplementary File S4. In the sixteen observational clinical studies, only two studies reported a sample size calculation, and one study reported that outcome assessors were blinded to the interventions.

None of the ten animal studies met all SYRCLE criteria. Most of the studies (80%) reported randomization without details, only one study reported the blinding of researchers, and half of the studies reported the blinding of outcome assessors during the study period, indicating risk of selection bias and performance bias. 

### 3.4. Biomarkers of Endothelial Dysfunction

Changes in biomarkers of endothelial activation and dysfunction over time during ECMO support are shown in Table 1 (A: patients; B: animals). The differences between ECMO and non-ECMO ill subjects are shown in Table 2 (A: patients; B: animals). The comparison of ECMO-supported subjects (healthy or ill) with healthy controls is summarized in Table 3 (A: patients; B: animals), and differences between patients with favorable and unfavorable outcomes are shown in Supplementary File S5.

#### 3.4.1. Syndecan-1

The endothelial glycocalyx is a thin, gel-like layer covering the luminal side of the vascular endothelium that forms a physical barrier between blood and endothelial cells [37]. It plays a crucial role in the regulation of vascular permeability, inflammation, coagulation and mechanotransduction [37]. Disruption of the endothelial glycocalyx is characterized by the shedding of its constituents, such as syndecan-1 or heparan sulfate, into the circulation. One patient [11] and two animal studies [29,35] investigated the course of circulating syndecan-1 and/or heparan sulfate during VA-ECMO. In adult patients undergoing lung transplantation supported by VA-ECMO during surgery, syndecan-1 levels measured immediately after lung transplantation were comparable to those in patients undergoing a lung transplantation without ECMO support, and remained stable till three days after transplantation [11]. In contrast, circulating syndecan-1 and heparan sulfate significantly increased over time in healthy dogs after six hours of VA-ECMO support [35]. Interestingly, this increase was less pronounced in dogs supported by ECMO with a pulsatile flow compared to a non-pulsatile flow [35]. Additionally, syndecan-1 levels significantly increased in rats with cardiac arrest on VA-ECMO support compared to baseline (before cardiac arrest) [29].

#### 3.4.2. Adhesion Molecules

Adhesion molecules and selectins mediate the rolling, activation and transendothelial migration of leukocytes [38]. The activated endothelium is characterized by the overexpression of adhesion molecules, such as intercellular adhesion molecule 1 (ICAM-1) and vascular cell adhesion molecule 1 (VCAM-1), as well as selectins such as P-selectin and E-selectin, and initiate a cascade of inflammatory mechanisms. Three patient [11,22,23] and two animal [33,36] studies on ICAM-1 and one patient study on VCAM-1 were included [23]. In lung transplant patients with intraoperative VA-ECMO, a notable reduction in ICAM-1 levels was observed 48 h post-ECMO compared to pre-transplant levels [11]. Conversely, in critically ill newborns undergoing VV- or VA-ECMO, ICAM-1 and VCAM-1 levels were comparable to those in healthy newborns [23], and ICAM-1 was not a predictor for more than 7 days of ECMO support [22]. Interestingly, heart tissue from rats with acute myocardial infarction supported by VA-ECMO showed increased myocardial ICAM-1 levels compared to healthy controls [33], while in rabbits with prolonged hemorrhagic shock, VA-ECMO resuscitation reduced the immunohistochemical staining of ICAM-1 expression in intestines compared to fluid resuscitation, but the levels were significantly higher here compared to healthy controls [36]. 

#### 3.4.3. Selectins

As for selectins, five studies were evaluated [18,20,22,24,34] including one animal study [34]. No significant alterations in E-selectin levels in neonates on VV-ECMO support were reported, whereas P-selectin levels progressively increased at the first day of VV-ECMO support [18]. In pediatric patients, E- and P-selectin levels did not differ between children on ECMO for primary respiratory failure nor for non-respiratory indications [20]. Moreover, E-selectin levels were significantly lower in children who died compared to those who survived when all ECMO types were taken together [20]. P-selectin level was not associated with prolonged ECMO assistance for more than seven days [22]. In respiratory failure patients on all types of ECMO support, there was a notable increase in E-selectin on both day 1 and day 7 during ECMO in patients with hemorrhagic complications compared to those without such complications, whereas elevated P-selectin levels were only observed on day 7 [24]. Furthermore, P-selectin levels were increased in pigs after cardiac arrest, while extracorporeal cardiopulmonary resuscitation diminished soluble P-selectin compared to conventional cardiopulmonary resuscitation following cardiac arrest [34].

#### 3.4.4. Soluble VE-Cadherin

Endothelial cells are connected by complex structures, including adherens junctions, which are mainly composed of VE-cadherin [39]. In response to inflammation, adherens junctions can be destabilized and this results in the increased shedding of VE-cadherin into the circulation, as well as increased endothelial permeability [39]. Only one rat study on VA-ECMO assistance, both with and without cardiac arrest, was included, and it showed higher levels of soluble VE-cadherin compared to healthy controls [32].

#### 3.4.5. Vascular Endothelial Growth Factor

Vascular Endothelial Growth Factor (VEGF) is a pro-inflammatory growth factor primarily known for its role in angiogenesis, but is also a key regulator of endothelial permeability [40]. Soluble VEGF was investigated in six studies [14,17,19,23,26,29]. In patients with ARDS, VEGF levels were comparable in patients on VV-ECMO support compared to patients without VV-ECMO support [17]. In these patients, soluble VEGF was higher compared to in healthy controls [17], whereas critically ill newborns supported by ECMO had lower VEGF levels than healthy controls [23]. Higher VEGF levels were found in children with sepsis on ECMO compared to those without ECMO support, irrespective of brain complications [19]. Interestingly, soluble VEGF levels decreased in adult ARDS patients in the first three days of VV-ECMO support [17], and in critically ill newborns on ECMO support [26], compared to patients without ECMO support. Additionally, non-survivors had lower VEGF levels compared to survivors in VA-ECMO patients [14], but not in ARDS patients on VV-ECMO support [17]. In rats with cardiac arrest on VA-ECMO, VEGF levels increased compared to levels before the induction of cardiac arrest [29].

#### 3.4.6. Angiopoietins

The endothelial angiopoietin/Tie2 system is one of the most important pathways involved in the regulation of endothelial permeability [41], and involves the regulatory interaction of the ligands angiopoietin-1 and angiopoietin-2 (ang-2) with the endothelial receptor Tie2. Especially, circulating ang-2 levels are associated with endothelial activation, injury severity, and worse clinical outcome in critically ill patients [42,43], and are therefore suggested as a marker of endothelial damage. Five patient studies and one rat study assessed this pathway [14,17,19,23,26,30]. In adult patients with ARDS, ang-2 levels were comparable between patients on ECMO support and those without ECMO, but were higher when compared to healthy controls [17]. Interestingly, ang-2 levels remained stable over time in ARDS patients supported by VV-ECMO, whereas ARDS patients without VV-ECMO showed a decrease in ang-2 levels over time [17]. Moreover, ang-2 levels were higher in non-survivors compared to survivors [17]. A similar effect was seen in critically ill adult patients on VA-ECMO support. Ang-2 levels remained stable over time; however, non-survivors had higher ang-2 levels compared to survivors [14]. Critically ill newborns supported by ECMO had increased ang-2 levels over time [26]. In addition, ang-2 levels were higher in critically ill newborn patients supported by ECMO compared to patients without ECMO support [26] as well as compared to healthy controls [23,26]. Moreover, the ang-2/ang-1 ratio was increased and soluble Tie2 levels were decreased in both children [19] and rats [30] with sepsis receiving ECMO support compared to no ECMO support.

#### 3.4.7. Von Willebrand Factor

Upon endothelial injury, von Willebrand factor (vWF) is released from the Weibel-Palade bodies into the circulation [44]. Three clinical studies explored vWF antigen levels [15,16,25], revealing stable vWF antigen levels over time in both VA- and VV-ECMO-supported patients with ARDS or cardiogenic shock, but high levels compared to normal assay levels [15,16]. In contrast, vWF antigen levels in adult severely ill patients decreased within 48 h on ECMO, but increased 24 h after weaning off ECMO compared to baseline when all ECMO types were taken together [25].

#### 3.4.8. Thrombomodulin

Thrombomodulin is a thrombin receptor expressed on endothelial cells and released after endothelial injury from the Weibel-Palade bodies [45]. Three clinical studies explored thrombomodulin levels [14,20,24]. In both adult and pediatric VV- or VA-ECMO patients, soluble thrombomodulin levels did not differ between survivors and non-survivors [14,20], but increased in ECMO-supported respiratory failure patients with hemorrhagic complications compared to those without such complications [24]. Interestingly, thrombomodulin levels were higher in children on ECMO for non-respiratory failure compared to those for primary respiratory indications [20].

#### 3.4.9. Extracellular Vesicles

One of the primary cellular responses to a damaged or compromised endothelium is the release of extracellular vesicles (EVs), making endothelial-derived EVs an early marker of endothelial dysfunction [46]. Four patient studies focused on EVs [12,13,22,23], with elevated endothelial-derived EV levels reported in both adult and pediatric VV- or VA-ECMO patients compared to healthy controls [13,21,23]. In contrast, endothelial-derived EVs decreased over time in adult cardiogenic shock patients on VA-ECMO [13]. A subsequent study showed that endothelial-derived EVs did not differ between survivors and non-survivors, determined at the first day of VA-ECMO support in critically ill patients [12]. Similarly, no significant differences were found between critically ill patients on VA-ECMO support compared to patients with ST-elevation myocardial infarction [12]. 

### 3.5. Microvascular Leakage and Edema Formation

A total of four animal studies investigated the consequences of endothelial activation and damage for endothelial permeability. Microvascular leakage can be assessed via dye-labeled macromolecule extravasation in different organs and protein content in bronchoalveolar lavage fluid (BALF). Edema formation is assessed by the organ wet-to-dry weight ratio. The comparison of animals on ECMO support with baseline measurements (pre-ECMO) is summarized in B in Table 1. The differences between ECMO and non-ECMO critically ill animals are shown in B in Table 2. B in Table 3 shows the differences between ECMO animal models and sham controls.

#### 3.5.1. Microvascular Leakage

One study assessed microvascular leakage through FITC-labeled albumin extravasation, in which rats on VA-ECMO support exhibited significant increased albumin extravasation in the capillaries of the mesentery compared to healthy controls [32]. Protein content in the BALF, as a marker of pulmonary capillary leakage, was measured in two studies, both revealing higher BALF protein levels in rats with lung injury on VV-ECMO support compared to healthy controls [27,28].

#### 3.5.2. Edema

Three preclinical studies investigated edema formation as assessed by lung organ wet-to-dry weight ratio [27,28,31]. Pulmonary edema was observed in rats with ARDS on VV-ECMO support compared to healthy controls [27,28], whereas ECMO seemed to reduce pulmonary edema when compared to those ARDS rats without ECMO [28]. In contrast, VA-ECMO in healthy rats resulted in pulmonary edema, which was worsened in diabetic rats on VA-ECMO support compared to healthy rats on ECMO support [31].

## 4. Discussion

ECMO is a life-saving intervention for patients with circulatory and/or pulmonary failure. However, the rate of complications remains high. ECMO induces a systemic inflammatory response, which may activate but also damage the endothelium, leading to increased vascular permeability and edema formation. To summarize the current evidence of the effects of ECMO on endothelial activation and damage, sixteen patient and ten animal studies were included.

The most often-studied endothelial activation markers were adhesion molecules (ICAM-1) and selectins (E- and P-selectin). The levels of endothelial activation markers were comparable to or higher than normal ranges. Compared to pre-ECMO or non-ECMO, most studies showed stable or decreased levels. Angiopoietin-2, von Willebrand Factor and extracellular vesicles were the most widely studied circulating markers of endothelial damage. Most of the included studies showed increased levels when compared to normal ranges, and pre-ECMO or non-ECMO values, suggesting (additional) endothelial damage. In healthy animals, ECMO itself leads to vascular leakage and edema. The effects of ECMO support in critically ill animals are contradictory. Taken together, this suggests that ECMO support (further) induces endothelial damage, but not endothelial activation, in the critically ill. Further research is necessary to conclude on the effect of the underlying comorbidity and type of ECMO support on endothelial dysfunction.

ECMO induces a systemic inflammatory response [2,3], which can activate the endothelium. In the current review, the most frequently investigated markers of endothelial activation were ICAM-1, P-selectin, and E-selectin. ECMO does not seem to activate the endothelium in critically ill patients, as evidenced by stable or decreasing levels over time or compared to non-ECMO patients. The possible reasons are that, in critically ill patients, the endothelium is already highly activated due to their underlying life-threatening illnesses, and that patients are in the recovery phase [41,47]. Despite its suggested association with various pathological conditions, endothelial activation presents a state of the endothelium to recruit leukocytes to the site of injury or infection, facilitating the immune response. Whether endothelial activation is associated with clinical outcomes is still unclear. In patients with sepsis, increased ICAM-1 and VCAM-1 levels have been associated with organ dysfunction and mortality [48,49]. In contrast, VCAM-1 was no longer independently associated with mortality after adjusting for inflammation in critically ill patients with systemic inflammatory response syndrome [50]. Taken together, these results suggest that ECMO support does not (further) activate the endothelium in the critically ill.

Endothelial damage can occur due to prolonged or severe endothelial activation, and is an important marker of poor outcomes in critical illness [51]. The included studies show that, following ECMO, there is more endothelial damage, as shown by increased circulating levels of ang-2 and vWF over time in critically ill patients, and elevated ang-2 in ECMO-supported patients compared to non-ECMO patients. Based on endothelial activation caused by the underlying disease, the use of ECMO seems to act as an extra hit, not further activating the endothelium but causing further damage to the endothelium. However, it is undeniable that the underlying disease in ECMO-assisted patients is also evolving all the time, and the observed findings may be the result of disease regression. In this regard, more evidence is needed. Interestingly, one study suggested that maintaining peak wall shear stress by pulsatility during ECMO could have protective effects on glycocalyx integrity [35]. Traditionally, ECMO generates non-pulsatile blood flow, which is non-physiological and may negatively impact endothelial integrity. However, the potential clinical effects with pulsatile flow remain inconclusive and limited [52]. Although evidence from CPB indicates that pulsatile flow may protect the endothelium [53], a large clinical study failed to demonstrate a definitive benefit in reducing stroke, mortality, or acute kidney injury [54]. This highlights the need for more research in this area. Furthermore, based on the comparisons between survivors and non-survivors, and patients with and without complications, ang-2 is the most often-studied marker, and seems to be associated with unfavorable outcomes [14,26], suggesting the ang/Tie2 pathway as a promising target of interest. Indeed, it was previously shown that pharmacologically targeting the angiopoietin/Tie2 system is promising in restoring the endothelium in critical illness [55,56]. In summary, the endothelium seems to be further damaged during ECMO support. More studies are needed that measure changes over time, not just at one time point, including time-points before and after ECMO initiation.

Endothelial damage can disrupt the integrity of the blood vessel wall and lead to vascular leakage and edema formation [57,58]. Animals connected to ECMO exhibited vascular leakage and edema, which was not only observed in critically ill animals [27,28], but also in healthy ones [31,32]. Although not always relatable to the human situation, the strength of animal studies lies in the possibility of differentiating between the effects of ECMO itself and the underlying disease. This is in alignment with the effect of CPB, which has also been shown to increase renal and pulmonary vascular leakage in healthy animals [55]. The possible reasons could include a systemic cytokine response caused by ECMO [32], or hemodilution [59]. The effects of ECMO on the endothelium in critically ill animals are very contrasting, which may be due to the underlying diseases or the type of ECMO support, and this requires further investigation. Taken together, ECMO itself seems to induce to vascular leakage and edema, whereas the effect of ECMO support in critically ill animals needs further investigation.

The current review has limitations. The effect of ECMO on endothelial function was not the primary objective of all included studies; therefore, these studies may not have been adequately powered for these analyses. Moreover, most included studies were relatively small single-center studies. The heterogeneity of the included studies makes this review susceptible to various forms of bias and various comparisons (such as changes over time, ECMO patients versus healthy controls, or non-ECMO patients, with or without complications), limiting the ability to perform meta-analyses. Despite the limitations, to the best of our knowledge, the current review provides the first overview of the published literature on the impact of ECMO on the endothelium. Moreover, not only clinical, but also animal studies were summarized, which strengthens our findings, as animal studies can exclusively investigate the effects of ECMO or the underlying disease.

This review outlines the current evidence on the course of ECMO-induced alterations in the endothelium. Further research is warranted to discover the full extent of ECMO-related endothelial dysfunction. For example, more studies are needed that measure changes over time, not just at one time point, including a time point before ECMO initiation. Furthermore, it is necessary to identify strategies to protect endothelial barrier function in critically ill patients, not only those on ECMO. Recent studies successfully proved the efficacy of agents that directly modulate the molecular systems involved in endothelial barrier regulation to reduce edema formation in multiple vital organs following CPB [55,60]. The strategies aimed directly at the endothelium, for example those targeting the ang/Tie2 system, appear to be one of the most promising strategies to reduce multiple vital organ injury and improve patient outcomes.

## 5. Conclusions

ECMO support seems to (further) cause endothelial damage, but not endothelial activation, in critically ill patients and animals. ECMO itself may lead to vascular leakage and edema in healthy animals. Further research is necessary to conclude on the effects of the underlying comorbidity and the type of ECMO support on endothelial dysfunction.

## Figures and Tables

**Table 1:** (**A**) Biomarkers of endothelial function in ECMO-supported patients over time. (**B**) Biomarkers of endothelial function in ECMO animal models over time.

	Study	Population	ECMO Indication	N	Type of ECMO	Day 1	Day 2	Day 3	Day 5	Post-ECMO
Syndecan-1	Coster et al. [11]	Adult	Lung transplant Intraoperative support	22	VA					<~>
ICAM-1	Coster et al. [11]	Adult	Lung transplant Intraoperative support	22	VA					<↓>
E-selectin	Cheung et al. [18]	Pediatric	Neonates with respiratory failure	10	VV	<~>				<~>
P-selectin	Cheung et al. [18]	Pediatric	Neonates with respiratory failure	10	VV	<↑>				<~>
VEGF	Patry et al. [17]	Adult	ARDS	16	VV	<↓>		<↓>		
	Rafat et al. [26]	Pediatric	Newborns with CDH	18	VA/VV	<↓>		<↓>		
Ang-2	Patry et al. [17]	Adult	ARDS	16	VV	<~>		<~>		
	Rafat et al. [26]	Pediatric	Newborns with CDH	18	VA/VV	<↑>		<↑>		
vWF	Mazzeffi et al. [15]	Adult	Cardiogenic shock	20	VA			<~>	<~>	
	Tauber et al. [25]	Adult	Cardiac/pulmonary failure	38	VA/VV	<↓>	<↓>			<↑>
EVs	Siegel et al. [13]	Adult	Cardiogenic shock	14	VA			<↓>		
	**Study**	**Species**	**ECMO Indication**		**N**	**Type of ECMO**		**6 h of ECMO vs. Baseline**		**Post-ECMO vs. Baseline**
Syndecan-1	Yin et al. [29]	Rat	ECPR		18	VA				↑
	Zhang et al. [35]	Dog	Cardiogenic shock		16	VA		↑		
Heparan sulfate	Zhang et al. [35]	Dog	Cardiogenic shock		16	VA		↑		
P-selectin	Liu et al. [34]	Pig	ECPR		10	VA				↑
VEGF	Yin et al. [29]	Rat	ECPR		18	VA				↑

<↓> represents a significant decrease in ECMO-supported patients compared to baseline measurement (pre-ECMO). <↑> represents a significant increase in ECMO-supported patients compared to baseline measurement. <~> represents no significant change compared to baseline value. Post-ECMO means measurement after weaning from ECMO. ECMO: Extracorporeal membrane oxygenation. VA: Veno-arterial. VV: Veno-venous. ICAM-1: Intercellular Adhesion Molecule 1. VEGF: Vascular Endothelial Growth Factor. Ang-2: Angiopoietin-2. vWF: Von Willebrand factor. EVs: Extracellular vesicles. ARDS: Acute Respiratory Distress Syndrome. CDH: Congenital Diaphragmatic Hernia. ↑ represents a significant increase in ECMO-supported animals compared to baseline measurement (pre-ECMO). Post-ECMO means after weaning from ECMO. ECMO: Extracorporeal membrane oxygenation. VA: Veno-arterial. ECPR: Extracorporeal cardiopulmonary resuscitation. VEGF: Vascular Endothelial Growth Factor.

**Table 2:** (**A**) Comparison of endothelial biomarkers in patients with and without ECMO support. (**B**) Comparison of endothelial biomarkers in animals with and without ECMO support.

(A)	Study	Population	Comparisons	N	Type of ECMO	Day 1	Day 7	Post-ECMO	ND
Syndecan-1	Coster et al. [11]	Adult	Lung transplant intraoperative ECMO vs. no support	22	VA			<~>	
ICAM-1	Coster et al. [11]	Adult	Lung transplant intraoperative ECMO vs. no support	22	VA			<~>	
VEGF	Patry et al. [17]	Adult	ARDS with ECMO vs. ARDS	16	VV				<~>
	Rafat et al. [26]	Pediatric	ECMO-dependent newborns with CDH vs. ECMO-independent	18	VA/VV				<↓>
	Xing et al. [19]	Pediatric	Severe sepsis with ECMO vs. simple pneumonia	13	VV		<↑>		
Ang-2	Patry et al. [17]	Adult	ARDS with ECMO vs. ARDS	16	VV				<~>
	Rafat et al. [26]	Pediatric	ECMO-dependent newborns with CDH vs. ECMO-independent	18	VA/VV				<↑>
Ang-2/Ang-1	Xing et al. [19]	Pediatric	Severe sepsis with ECMO vs. simple pneumonia	13	VV		<↑>		
sTie2	Xing et al. [19]	Pediatric	Severe sepsis with ECMO vs. simple pneumonia	13	VV		<↓>		
EVs	Siegel et al. [12]	Adult	Cardiogenic shock with ECMO vs. STEMI	18	VA	<~>			
**(B)**	**Study**		**Species**	**Comparisons**		**N**	**Type of ECMO**	**Difference**	
ICAM-1 expression (intestine)	Zhao et al. [36]		Rabbit	Hemorrhagic shock + ECMO vs. hemorrhagic shock		10	VA	↓	
P-selectin	Liu et al. [34]		Pig	ECPR vs. CCPR		10	VA	↓	
Ang-2/Ang-1	Xing et al. [30]		Rat	Sepsis + ECMO vs. sepsis		5	VA	↑	
sTie2	Xing et al. [30]		Rat	Sepsis + ECMO vs. sepsis		5	VA	↓	
BALF protein	Huang et al. [28]		Rat	Acute lung injury + ECMO vs. acute lung injury		6	VV	↓	
Wet/dry ratio(lung)	Huang et al. [28]		Rat	Acute lung injury + ECMO vs. acute lung injury		6	VV	↓	

<↓> represents a significant decrease in ECMO-supported patients compared to the non-ECMO group. <↑> represents a significant increase in ECMO-supported patients compared to non-ECMO group. <~> represents no significant change compared to non-ECMO group. Post-ECMO means time after weaning from ECMO. ND means not determined. ECMO: Extracorporeal membrane oxygenation. VA: Veno-arterial. VV: Veno-venous. ARDS: Acute Respiratory Distress Syndrome. CDH: Congenital Diaphragmatic Hernia. STEMI: ST-elevation myocardial infarction. ICAM-1: Intercellular Adhesion Molecule 1, VEGF: Vascular Endothelial Growth Factor. Ang-2: Angiopoietin-2. sTie2: Soluble Tie2. EVs: Extracellular vesicles. ECPR: Extracorporeal cardiopulmonary resuscitation. ↑ represents a significant increase in ECMO-supported animals compared to non-ECMO group. ↓ represents a significant decrease in ECMO-supported animals compared to non-ECMO Group. CCPR: Conventional cardiopulmonary resuscitation. BALF: Bronchoalveolar lavage fluid.

**Table 3:** (**A**) Comparisons of endothelial function biomarkers in ECMO-supported patients vs. healthy controls. (**B**) Comparisons of endothelial biomarkers in ECMO-supported animal models vs. sham controls.

(A)	Study	Population	ECMO Indication	N	Type of ECMO	Day 0		Day 1	Day 3	Day 5	Day 7	Post-ECMO	ND
ICAM-1	Vítková et al. [23]	Pediatric	Critically ill newborns	13	VA/VV								<~>
VCAM-1	Vítková et al. [23]	Pediatric	Critically ill newborns	13	VA/VV								<~>
VEGF	Patry et al. [17]	Adult	ARDS	16	VV								<↑>
	Rafat et al. [26]	Pediatric	Newborns with CDH	18	VA/VV								<↓>
	Vítková et al. [23]	Pediatric	Critically ill newborns	13	VA/VV								<↓>
Ang-2	Patry et al. [17]	Adult	ARDS	16	VV								<↑>
	Rafat et al. [26]	Pediatric	Newborns with CDH	18	VA/VV								<↑>
	Vítková et al. [23]	Pediatric	Critically ill newborns	13	VA/VV								<↑>
vWF*	Hékimian et al. [16]	Adult	ARDS	30	VV	<↑>					<↑>		
	Mazzeffi et al. [15]	Adult	Cardiogenic shock	20	VA			<↑>	<↑>	<↑>			
	Tauber et al. [25]	Adult	Cardiac/pulmonary failure	38	VA/VV	<↑>		<↑>				<↑>	
EVs	Chandler et al. [21]	Pediatric	Cardiac/pulmonary failure	55	VA/VV			<↑>					
	Siegel et al. [13]	Adult	Cardiogenic shock	14	VA			<↑>	<↑>			<↑>	
	Vítková et al. [23]	Pediatric	Critically ill newborns	13	VA/VV								<↑>
**(B)**		**Study**	**Species**	**Comparison**					**N**	**Type of ECMO**		**Difference**	
ICAM-1 (heart tissue)		Cheng et al. [33]	Rat	Acute myocardial infarction + ECMO vs. sham control					6	VA		↑	
ICAM-1 expression (intestine)		Zhao et al. [36]	Rabbit	Hemorrhagic shock + ECMO vs. sham control					10	VA		↑	
Soluble VE-Cadherin		Wollborn et al. [32]	Rat	Healthy ECMO vs. sham control or ECPR vs. sham control					14	VA		↑	
FITC-albumin		Wollborn et al. [32]	Rat	Healthy ECMO vs. sham control or ECPR vs. sham control					14	VA		↑	
BALF protein		Huang et al. [28]	Rat	Acute lung injury + ECMO vs. sham control					6	VV		↑	
		Zhang et al. [27]	Rat	ARDS + ECMO vs. sham control					10	VV		↑	
Wet/dry ratio(lung)		Fujii et al. [31]	Rat	Healthy ECMO vs. sham control					14	VA		↑	
		Huang et al. [28]	Rat	Acute lung injury + ECMO vs. sham control					6	VV		↑	
		Zhang et al. [27]	Rat	ARDS + ECMO vs. sham control					10	VV		↑	

<↓> represents a significant decrease in ECMO-supported patients compared to normal range or values in healthy controls. <↑> represents a significant increase in ECMO-supported patients compared to normal range or values in healthy controls. <~> represents no significant change compared to normal range or values in healthy controls. Post-ECMO means time after weaning from ECMO. ND means not determined. * compared to normal ranges. ECMO: Extracorporeal membrane oxygenation. VA: Veno-arterial. VV: Veno-venous. ARDS: Acute Respiratory Distress Syndrome. CDH: Congenital Diaphragmatic Hernia. ICAM-1: Intercellular Adhesion Molecule 1. VCAM-1: Vascular Cell Adhesion Molecule 1. VEGF: Vascular Endothelial Growth Factor., Ang-2: Angiopoietin-2. vWF: von Willebrand Factor. EVs: Extracellular vesicles. ↑ represents a significant increase in ECMO-supported animals compared to sham controls. ECPR: Extracorporeal cardiopulmonary resuscitation. VE-Cadherin: Vascular Endothelial Cadherin. FITC: Fluorescein Isothiocyanate. BALF: Bronchoalveolar lavage fluid.

## Supplementary Materials

The following supporting information can be downloaded at: https://www.mdpi.com/article/10.3390/ijms251910680/s1.
