# Supplementary material for: Exploring the Impact of Extracorporeal Membrane Oxygenation on the Endothelium: A Systematic Review
Source: Int J Mol Sci. 2024 Oct 3;25(19):10680. doi: 10.3390/ijms251910680 (PMC11477268; doi:10.3390/ijms251910680)
Supplement: Supplementary file 1 [file ijms-25-10680-s001.zip › Supplementary File S1.pdf]

Supplementary file S1: Search strategy

22-2-2023:

Databases:		
PubMed, Embase (Ovid)	Before deduplication	After deduplication
Total	1145	766

PUBMED
448 hits
(("Extracorporeal Membrane Oxygenation"[Mesh] OR extracorporeal membrane oxygenat*[tiab] OR extra-corporeal membrane oxygenat*[tiab] OR ECMO[tiab] OR extracorporeal life support[tiab] OR ECLS[tiab] OR Venoarterial ECMO[tiab] OR Venoarterial Extracorporeal Membrane Oxygenat*[tiab] OR veno-arterial ECMO[tiab] OR VA ECMO[tiab] OR Venovenous ECMO[tiab] OR Venovenous Extracorporeal Membrane Oxygenat*[tiab] OR veno-venous ECMO[tiab] OR VV ECMO[tiab])
AND
("Endothelium, Vascular"[Mesh] OR "Glycocalyx"[Mesh] OR endothel*[tiab] OR endothelial activat*[tiab] OR endothelial damage*[tiab] OR glycocalyx[tiab] OR "Capillary Fragility"[Mesh] OR "Capillary Permeability"[Mesh] OR "Capillary Leak Syndrome"[Mesh] OR edema*[tiab] OR oedema*[tiab] OR capillary leak*[tiab] OR vascular leak*[tiab] OR microvascular leak*[tiab] OR capillary permeab*[tiab] OR vascular permeab*[tiab] OR microvascular permeab*[tiab] OR capillary barrier*[tiab] OR vascular barrier*[tiab] OR microvascular barrier*[tiab] OR fluid extravasat*[tiab] OR capillary hyperpermeab*[tiab] OR vascular hyperpermeab*[tiab] OR microvascular hyperpermeab*[tiab] OR capillary hyper-permeab*[tiab] OR vascular hyper-permeab*[tiab] OR microvascular hyper-permeab*[tiab] OR capillary fragilit*[tiab] OR endothelial fragilit*[tiab] OR vascular fragilit*[tiab] OR microvascular fragilit*[tiab]))
NOT
("Congress" [Publication Type] OR "Case Reports" [Publication Type] OR "Comment" [Publication Type] OR "Editorial" [Publication Type] OR "Letter" [Publication Type] OR case report[ti] OR letter[ti] OR comment*[ti])

EMBASE (via Ovid):
Database(s): Embase Classic+Embase 1947 to 2023 February 21
Search Strategy:

#	Searches	Results
1	exp extracorporeal oxygenation/	40213
2	(extracorporeal membrane oxygenat* or extra-corporeal membrane oxygenat* or ECMO or extracorporeal life support or ECLS or Venoarterial ECMO or Venoarterial	37337

	Extracorporeal Membrane Oxygenat* or veno-arterial ECMO or VA ECMO or Venovenous ECMO or Venovenous Extracorporeal Membrane Oxygenat* or veno-venous ECMO or VV ECMO).ti,ab,kf.	
3	1 or 2	50628
4	exp vascular endothelium/ or glycocalyx/ or vascular fragility/ or capillary permeability/ or capillary leak syndrome/	93288
5	(endothel* or endothelial activat* or endothelial damage* or glycocalyx or edema* or oedema* or capillary leak* or vascular leak* or microvascular leak* or capillary permeab* or vascular permeab* or microvascular permeab* or capillary barrier* or vascular barrier* or microvascular barrier* or fluid extravasat* or capillary hyperpermeab* or vascular hyperpermeab* or microvascular hyperpermeab* or capillary hyper-permeab* or vascular hyper-permeab* or microvascular hyper-permeab* or capillary fragilit* or endothelial fragilit* or vascular fragilit* or microvascular fragilit*).ti,ab,kf.	845873
6	4 or 5	862027
7	3 and 6	1945
8	limit 7 to conference abstract status	875
9	7 not 8	1070
10	exp conference paper/ or editorial/ or exp erratum/ or letter/ or note/ or case report/ or (letter or editorial or comment* or case report).ti.	8256248
11	9 not 10	697

UPDATE SEARCH: 22-2-2023 t/m 3-11-2023

3-11-2023:

Databases:		
PubMed, Embase (Ovid)	Before deduplication	After deduplication
Total	113	64

<u>PUBMED</u>

47 hits

(("Extracorporeal Membrane Oxygenation"[Mesh] OR extracorporeal membrane oxygenat*[tiab] OR extra-corporeal membrane oxygenat*[tiab] OR ECMO[tiab] OR extracorporeal life support[tiab] OR ECLS[tiab] OR Venoarterial ECMO[tiab] OR Venoarterial Extracorporeal Membrane Oxygenat*[tiab] OR veno-arterial ECMO[tiab] OR VA ECMO[tiab] OR Venovenous ECMO[tiab] OR Venovenous Extracorporeal Membrane Oxygenat*[tiab] OR veno-venous ECMO[tiab] OR VV

ECMO[tiab])
AND
("Endothelium, Vascular"[Mesh] OR "Glycocalyx"[Mesh] OR endothel*[tiab] OR endothelial activat*[tiab] OR endothelial damage*[tiab] OR glycocalyx[tiab] OR "Capillary Fragility"[Mesh] OR "Capillary Permeability"[Mesh] OR "Capillary Leak Syndrome"[Mesh] OR edema*[tiab] OR oedema*[tiab] OR capillary leak*[tiab] OR vascular leak*[tiab] OR microvascular leak*[tiab] OR capillary permeab*[tiab] OR vascular permeab*[tiab] OR microvascular permeab*[tiab] OR capillary barrier*[tiab] OR vascular barrier*[tiab] OR microvascular barrier*[tiab] OR fluid extravasat*[tiab] OR capillary hyperpermeab*[tiab] OR vascular hyperpermeab*[tiab] OR microvascular hyperpermeab*[tiab] OR capillary hyper-permeab*[tiab] OR vascular hyper-permeab*[tiab] OR microvascular hyper-permeab*[tiab] OR capillary fragilit*[tiab] OR endothelial fragilit*[tiab] OR vascular fragilit*[tiab] OR microvascular fragilit*[tiab]))
NOT
("Congress" [Publication Type] OR "Case Reports" [Publication Type] OR "Comment" [Publication Type] OR "Editorial" [Publication Type] OR "Letter" [Publication Type] OR case report[ti] OR letter[ti] OR comment*[ti])
AND ("2023/2/22"[Date - Publication] : "2023/11/3"[Date - Publication])

EMBASE (via Ovid):
Database(s): Embase Classic+Embase 1947 to 2023 November 02
Search Strategy:

#	Searches	Results
1	exp extracorporeal oxygenation/	43263
2	(extracorporeal membrane oxygenat* or extra-corporeal membrane oxygenat* or ECMO or extracorporeal life support or ECLS or Venoarterial ECMO or Venoarterial Extracorporeal Membrane Oxygenat* or veno-arterial ECMO or VA ECMO or Venovenous ECMO or Venovenous Extracorporeal Membrane Oxygenat* or veno-venous ECMO or VV ECMO).ti,ab,kf.	38913
3	1 or 2	53853
4	exp vascular endothelium/ or glycocalyx/ or vascular fragility/ or capillary permeability/ or capillary leak syndrome/	94698
5	(endothel* or endothelial activat* or endothelial damage* or glycocalyx or edema* or oedema* or capillary leak* or vascular leak* or microvascular leak* or capillary permeab* or vascular permeab* or microvascular permeab* or capillary barrier* or vascular barrier* or microvascular barrier* or fluid extravasat* or capillary hyperpermeab* or vascular hyperpermeab* or microvascular hyperpermeab* or capillary hyper-permeab* or vascular hyper-permeab* or microvascular hyper-permeab* or capillary fragilit* or endothelial fragilit* or vascular fragilit* or	860445

	microvascular fragilit*).ti,ab,kf.	
6	4 or 5	877019
7	3 and 6	2067
8	limit 7 to conference abstract status	959
9	7 not 8	1108
10	exp conference paper/ or editorial/ or exp erratum/ or letter/ or note/ or case report/ or (letter or editorial or comment* or case report).ti.	8589339
11	9 not 10	719
12	limit 11 to yr="2023 -Current"	66

UPDATE SEARCH 9-4-2024

9-4-2024:

Databases:		
PubMed, Embase (Ovid)	Before deduplication	After deduplication* Met de 2 oudere files dd 22-2-2023 en 3-11-2023
Total	1252	45

PUBMED

498 hits

(("Extracorporeal Membrane Oxygenation"[Mesh] OR extracorporeal membrane oxygenat*[tiab] OR extra-corporeal membrane oxygenat*[tiab] OR ECMO[tiab] OR extracorporeal life support[tiab] OR ECLS[tiab] OR Venoarterial ECMO[tiab] OR Venoarterial Extracorporeal Membrane Oxygenat*[tiab] OR veno-arterial ECMO[tiab] OR VA ECMO[tiab] OR Venovenous ECMO[tiab] OR Venovenous Extracorporeal Membrane Oxygenat*[tiab] OR veno-venous ECMO[tiab] OR VV ECMO[tiab])

AND

("Endothelium, Vascular"[Mesh] OR "Glycocalyx"[Mesh] OR endothel*[tiab] OR endothelial activat*[tiab] OR endothelial damage*[tiab] OR glycocalyx[tiab] OR "Capillary Fragility"[Mesh] OR "Capillary Permeability"[Mesh] OR "Capillary Leak Syndrome"[Mesh] OR edema*[tiab] OR oedema*[tiab] OR capillary leak*[tiab] OR vascular leak*[tiab] OR microvascular leak*[tiab] OR capillary permeab*[tiab] OR vascular permeab*[tiab] OR microvascular permeab*[tiab] OR capillary barrier*[tiab] OR vascular barrier*[tiab] OR microvascular barrier*[tiab] OR fluid extravasat*[tiab] OR capillary hyperpermeab*[tiab] OR vascular hyperpermeab*[tiab] OR microvascular hyperpermeab*[tiab] OR capillary hyper-permeab*[tiab] OR vascular hyper-permeab*[tiab] OR microvascular hyper-permeab*[tiab] OR capillary fragilit*[tiab] OR endothelial fragilit*[tiab] OR

vascular fragilit*[tiab] OR microvascular fragilit*[tiab]))
NOT
("Congress" [Publication Type] OR "Case Reports" [Publication Type] OR "Comment" [Publication Type] OR "Editorial" [Publication Type] OR "Letter" [Publication Type] OR case report[ti] OR letter[ti] OR comment*[ti])

EMBASE (via Ovid):
Database(s): **Embase Classic+Embase** 1947 to 2024 April 08
Search Strategy:

#	Searches	Results
1	exp extracorporeal oxygenation/	46204
2	(extracorporeal membrane oxygenat* or extra-corporeal membrane oxygenat* or ECMO or extracorporeal life support or ECLS or Venoarterial ECMO or Venoarterial Extracorporeal Membrane Oxygenat* or veno-arterial ECMO or VA ECMO or Venovenous ECMO or Venovenous Extracorporeal Membrane Oxygenat* or veno-venous ECMO or VV ECMO).ti,ab,kf.	41011
3	1 or 2	56893
4	exp vascular endothelium/ or glycocalyx/ or vascular fragility/ or capillary permeability/ or capillary leak syndrome/	96209
5	(endothel* or endothelial activat* or endothelial damage* or glycocalyx or edema* or oedema* or capillary leak* or vascular leak* or microvascular leak* or capillary permeab* or vascular permeab* or microvascular permeab* or capillary barrier* or vascular barrier* or microvascular barrier* or fluid extravasat* or capillary hyperpermeab* or vascular hyperpermeab* or microvascular hyperpermeab* or capillary hyper-permeab* or vascular hyper-permeab* or microvascular hyper-permeab* or capillary fragilit* or endothelial fragilit* or vascular fragilit* or microvascular fragilit*).ti,ab,kf.	877097
6	4 or 5	893911
7	3 and 6	2191
8	limit 7 to conference abstract status	1023
9	7 not 8	1168
10	exp conference paper/ or editorial/ or exp erratum/ or letter/ or note/ or case report/ or (letter or editorial or comment* or case report).ti.	8850916
11	9 not 10	754
