# Supplementary material for: Exploring the Impact of Extracorporeal Membrane Oxygenation on the Endothelium: A Systematic Review
Source: Int J Mol Sci. 2024 Oct 3;25(19):10680. doi: 10.3390/ijms251910680 (PMC11477268; doi:10.3390/ijms251910680)
Supplement: Supplementary file 1 [file ijms-25-10680-s001.zip › Supplementary File S2.pdf]

Supplementary file S2: Characteristics of included studies investigating endothelial biomarkers in patients on ECMO support

Author, Year	Country	Study design	Population	Study groups	No. of ECMO Subjects	Type of ECMO	Indication of ECMO	Main finding
Caprarola, 2022	USA	Retrospective observational	Pediatric	Respiratory vs. non-respiratory; survivor vs. non-survivor; normal vs. abnormal neuroimaging	99	VA/VV	All possible indications	High levels of circulating inflammatory, endothelial activation, and fibrinolytic markers are associated with mortality and abnormal neuroimaging in pediatric on ECMO.
Chandler, 2021	USA	Prospective observational	Pediatric	NA	55	VA/VV	All possible indications	ECMO patients had two- to threefold higher levels of EVs

								compared with normal range.
Cheung, 2000	Canada	Prospective observational	Pediatric	NA	10	VV	Severe respiratory failure	ECMO resulted in a time-dependent increase in plasma soluble P-selectin concentration.
Coster, 2023	USA	Retrospective observational	Adult	No-Support vs. VA-ECMO vs. CPB	22	VA	Intraoperative use in lung transplantation	Higher plasma concentrations of endothelial injury biomarkers are associated with CPB compared to VA-ECMO or no support.
Hékimian, 2021	France	Prospective observational	Adult	Non-COVID-19 vs. COVID-19	30	VV	ARDS	VWF: Ag levels were fourfold higher than the upper limit of the normal

								range in patients on VV-ECMO.
Jang, 2023	Republic of Korea	Retrospective observational	Adult	With vs. without hemorrhagic complications	132	VA/VV/VAV	Respiratory failure	Initial TNF-α, tissue factor, soluble thrombomodulin, E-selectin, and activated protein C levels were significantly associated with hemorrhagic complications.
Mazzeffi, 2019	USA	Prospective observational	Adult	NA	20	VA	Cardiogenic shock	In VA-ECMO patients, VWF: Ag levels were high compared to values in healthy controls and remained high within the first 5 days after initiation of

								ECMO.
Pais, 2020	UK	Prospective observational	Pediatric	ECMO7+d vs. ECMO<7d	24	VA/VV	Persistent pulmonary hypertension of the newborn	There was no difference between groups (ECMO 7+d versus ECMO<7d) for P-selectin and ICAM-1 levels.
Patry, 2020	Germany	Prospective observational	Adult	ECMO vs. non-ECMO vs. healthy control; Survivors vs. non-survivors	16	VV	ARDS	During ECMO, VEGF serum level declined significantly while Ang-2 serum levels in non-survivors were significantly increased compared to survivors.

Rafat, 2019	Germany	Prospective observational	Pediatric	ECMO-dependent vs. ECMO-independent vs. healthy control	18	VA/VV	Congenital diaphragmatic hernia	ECMO support in newborns with congenital diaphragmatic hernia is associated with decreased VEGF and increased Ang-2 serum levels.
Siegel, 2021	Germany	Prospective observational	Adult	VA-ECMO vs. STEMI; Survivors vs. non-survivors	18	VA	Cardiogenic shock/ECPR	Endothelial-derived EVs did not differ between survivors and non-survivors on VA-ECMO support.
Siegel, 2020	Germany	Prospective observational	Adult	Survivors vs. non-survivors; ECMO vs. healthy control	14	VA	Cardiogenic shock/ECPR	Patients on VA-ECMO had increased levels of endothelial-derived EVs after ECMO

								initiation compared to healthy controls.
Tauber, 2015	Austria	Prospective observational	Adult	VA- vs. VV-ECMO	38	VA/VV	Cardiac and/or pulmonary failure	Compared with baseline measurements, vWF:Ag decreased after 24 hours of ECMO support and remained lower during ongoing support. After patients were weaned from ECMO support, vWF:Ag increased above baseline.
Tsai, 2019	China Taiwan	Prospective observational	Adult	Survivors vs. non-survivors	23	VA	Cardiogenic shock	Combination of Ang-2 at day 0 and VEGF at day 2 showed a

								modest performance on mortality discrimination in patients on ECMO.
Vítková, 2018	Czech Republic	Prospective observational	Pediatric	ECMO vs. healthy control	13	VA/VV	All possible indications	The assessment of endothelial markers showed significantly higher concentration of Ang-2 and lower concentration of VEGF in ECMO group.
Xing, 2021	China	Prospective observational	Pediatric	ECMO support with vs. without brain complication vs. non-ECMO support simple pneumonia	13	VV	Pneumonia/sepsis	During ECMO an increased Ang-2/Ang-1 level and a decreased sTie2 were consistent with severe

clinical outcomes in the brain.

NA not applicable, *ECMO* extracorporeal membrane oxygenation, *VA* veno-arterial, *VV* veno-venous, *VAV* venoarterial-venous, *CPB* cardiopulmonary bypass, *STEMI* ST-elevation myocardial infarction, *ARDS* acute respiratory distress syndrome, *ECPR* extracorporeal cardiopulmonary resuscitation, *EVs* extracellular vesicles, *VWF: Ag* von Willebrand factor antigen, *ICAM-1* intercellular adhesion molecule 1, *VEGF* vascular endothelial growth factor, *TNF-α* tumor necrosis factor alpha, *TF* tissue factor, *Ang-2* Angiopoietin 2, *Ang-1* Angiopoietin 1, *sTie2* soluble Tie2
