# Supplementary material for: Exploring the Impact of Extracorporeal Membrane Oxygenation on the Endothelium: A Systematic Review
Source: Int J Mol Sci. 2024 Oct 3;25(19):10680. doi: 10.3390/ijms251910680 (PMC11477268; doi:10.3390/ijms251910680)
Supplement: Supplementary file 1 [file ijms-25-10680-s001.zip › Supplementary File S3.pdf]

Supplementary file S3: Animal characteristics and experimental protocols on ECMO

Species	Studies (number)	Sex (m/f/nd)	Group size (number)	Weight range (kg)	Type of ECMO (VA/VV)	Indication of ECMO	Priming volume (ml)	Flow range (mL/kg/ min)	Time of ECMO support (h)
Rat	7	6/1/0	4-10	0.22-0.5	5/2	ARDS(2)/ cardiac arrest(2)/ sepsis(1)/ /AMI(1)/no indication(1)	6-20.3	40-150	0.5-3
Pig	1	1/0/0	10	35.13±5. 57	1/0	Cardiac arrest	nd	50-nd	After ROSC
Dog	1	0/0/1	8	10	1/0	Cardiac shock	200	130	6
Rabbit	1	1/0/0	10	2.3-2.6	1/0	Hemorrhag ic shock	50	<50	2

nd not determined, *ECMO* extracorporeal membrane oxygenation, *VA* veno-arterial, *VV* veno-venous, *ARDS* acute respiratory distress syndrome, *AMI* acute myocardial infarction, *ROSC* return of spontaneous circulation
