# Supplementary material for: Exploring the Impact of Extracorporeal Membrane Oxygenation on the Endothelium: A Systematic Review
Source: Int J Mol Sci. 2024 Oct 3;25(19):10680. doi: 10.3390/ijms251910680 (PMC11477268; doi:10.3390/ijms251910680)
Supplement: Supplementary file 1 [file ijms-25-10680-s001.zip › Supplementary File S4.pdf]

Supplementary file S4: Quality assessment of included studies

Supplementary Table 1. Individual SYRCLE Risk of Bias assessment for *in vivo* studies with explanations

**Study**	**Sequence generation (selection bias)**	**Baseline characteristics (selection bias)**	**Allocation concealment (selection bias)**	**Random housing (performance bias)**	**Blinding (performance bias)**	**Random outcome assessment (detection bias)**	**Blinding (detection bias)**	**Incomplete outcome data (attrition bias)**	**Selective outcome reporting (reporting bias)**	**Other source of bias (other)**
Zhang, 2022	Unclear	Yes	Unclear	Unclear	No	Yes	Yes	Unclear	Yes	Yes
Huang, 2022	No	Unclear	Unclear	Unclear	No	Yes	Unclear	Unclear	Yes	Unclear
Zhang, 2021	Unclear	Yes	Unclear	Unclear	No	Unclear	No	Unclear	Yes	Yes
Yin, 2021	Unclear	Yes	Unclear	Yes	No	No	Yes	Yes	Yes	Yes
Xing, 2021	Unclear	Unclear	Unclear	Unclear	No	No	No	Unclear	Yes	Yes
Fujii, 2021	No	Yes	Unclear	Yes	No	Unclear	Unclear	Unclear	Yes	Yes
Wollborn, 2019	Unclear	Unclear	Unclear	Unclear	Yes	Yes	Yes	Unclear	Yes	Yes
Liu, 2019	Unclear	Yes	Unclear	Yes	No	Unclear	Yes	Unclear	Yes	Yes
Cheng, 2022	Unclear	Yes	Unclear	Yes	No	Unclear	Unclear	Unclear	Yes	Yes
Zhao, 2014	Unclear	Yes	Unclear	Yes	No	Unclear	Yes	Unclear	Yes	Yes

"Yes", "Unclear", "No" indicates "low", "unclear", and "high" risk of bias, respectively

Item	Type of bias	Domain	Description of domain	Review authors judgment
1	Selection bias	Sequence generation	Describe the methods used, if any, to generate the allocation sequence in sufficient detail to allow an assessment whether it should produce comparable groups.	Was the allocation sequence adequately generated and applied? (*)
2	Selection bias	Baseline characteristics	Describe all the possible prognostic factors or animal characteristics, if any, that are compared in order to judge whether or not intervention and control groups were similar at the start of the experiment.	Were the groups similar at baseline or were they adjusted for confounders in the analysis?
3	Selection bias	Allocation concealment	Describe the method used to conceal the allocation sequence in sufficient detail to determine whether intervention allocations could have been foreseen before or during enrolment.	Was the allocation adequately concealed? (*)
4	Performance bias	Random housing	Describe all measures used, if any, to house the animals randomly within the animal room.	Were the animals randomly housed during the experiment?

Item	Type of bias	Domain	Description of domain	Review authors judgment
5	Performance bias	Blinding	Describe all measures used, if any, to blind trial caregivers and researchers from knowing which intervention each animal received. Provide any information relating to whether the intended blinding was effective.	Were the caregivers and/or investigators blinded from knowledge which intervention each animal received during the experiment?
6	Detection bias	Random outcome assessment	Describe whether or not animals were selected at random for outcome assessment, and which methods to select the animals, if any, were used.	Were animals selected at random for outcome assessment?
7	Detection bias	Blinding	Describe all measures used, if any, to blind outcome assessors from knowing which intervention each animal received. Provide any information relating to whether the intended blinding was effective.	Was the outcome assessor blinded?
8	Attrition bias	Incomplete outcome data	Describe the completeness of outcome data for each main outcome, including attrition and exclusions from the analysis. State whether attrition and exclusions were reported, the numbers in each intervention	Were incomplete outcome data adequately addressed? (*)

Item	Type of bias	Domain	Description of domain	Review authors judgment
			group (compared with total randomized animals), reasons for attrition or exclusions, and any re-inclusions in analyses for the review.	
9	Reporting bias	Selective outcome reporting	State how selective outcome reporting was examined and what was found.	Are reports of the study free of selective outcome reporting? (*)
10	Other	Other sources of bias	State any important concerns about bias not covered by other domains in the tool.	Was the study apparently free of other problems that could result in high risk of bias? (*)

*Items in agreement with the items in the Cochrane Risk of Bias tool.

Supplementary Table 2 Quality assessment of included clinical observational studies

**Study**	**1**	**2**	**3**	**4**	**5**	**6**	**7**	**8**	**9**	**10**	**11**	**12**	**13**	**14**
Coster, 2023	No	Yes	CD	Yes	No	Yes	Yes	NA	Yes	NA	Yes	NR	Yes	Yes
Caprarola,2022	Yes	Yes	Yes	Yes	No	Yes	Yes	NA	Yes	NA	Yes	Yes	CD	NR
Xing, 2021	No	Yes	CD	Yes	No	Yes	Yes	NA	Yes	NA	CD	NR	Yes	NR
Siegel, 2021	Yes	Yes	Yes	No	No	Yes	Yes	NA	Yes	NA	Yes	NR	Yes	NR
Hékimian, 2021	Yes	Yes	CD	Yes	No	Yes	Yes	NA	Yes	NA	Yes	NR	Yes	NR
Chandler, 2021	Yes	No	CD	CD	No	Yes	Yes	NA	No	NA	Yes	NR	CD	NR
Siegel, 2020	Yes	Yes	CD	Yes	No	Yes	Yes	NA	Yes	NA	Yes	NR	Yes	NR
Patry, 2020	Yes	Yes	CD	Yes	No	Yes	Yes	NA	Yes	NA	Yes	NR	Yes	NR
Tsai, 2019	Yes	Yes	No	Yes	No	Yes	Yes	NA	Yes	NA	Yes	NR	Yes	NR
Rafat, 2019	Yes	Yes	CD	Yes	No	Yes	Yes	NA	Yes	NA	Yes	NR	Yes	NR
Pais, 2020	Yes	Yes	No	Yes	No	Yes	Yes	NA	Yes	NA	Yes	NR	Yes	Yes
Vítková, 2018	Yes	Yes	CD	No	No	Yes	Yes	NA	Yes	NA	Yes	NR	Yes	NR
Cheung, 2000	Yes	Yes	CD	Yes	No	Yes	Yes	NA	Yes	NA	Yes	NR	Yes	NR
Mazzeffi, 2019	Yes	No	CD	CD	Yes	Yes	Yes	NA	Yes	NA	Yes	NR	Yes	NR
Jang, 2023	Yes	Yes	Yes	Yes	No	Yes	Yes	NA	Yes	NA	Yes	NR	Yes	Yes
Tauber, 2015	Yes	Yes	CD	Yes	Yes	Yes	Yes	NA	Yes	NA	Yes	NR	Yes	NR

*CD, cannot determine; NA, not applicable; NR, not reported

1. Was the research question or objective in this paper clearly stated?
2. Was the study population clearly specified and defined?
3. Was the participation rate of eligible persons at least 50%?
4. Were all the subjects selected or recruited from the same or similar populations (including the same time period)? Were inclusion and exclusion criteria for being in the study prespecified and applied uniformly to all participants?

5. Was a sample size justification, power description, or variance and effect estimates provided?
6. For the analyses in this paper, were the exposure(s) of interest measured prior to the outcome(s) being measured?
7. Was the timeframe sufficient so that one could reasonably expect to see an association between exposure and outcome if it existed?
8. For exposures that can vary in amount or level, did the study examine different levels of the exposure as related to the outcome (e.g., categories of exposure, or exposure measured as continuous variable)?
9.Were the exposure measures (independent variables) clearly defined, valid, reliable, and implemented consistently across all study participants?
10. Was the exposure(s) assessed more than once over time?
11. Were the outcome measures (dependent variables) clearly defined, valid, reliable, and implemented consistently across all study participants?
12. Were the outcome assessors blinded to the exposure status of participants?
13. Was loss to follow-up after baseline 20% or less?
14. Were key potential confounding variables measured and adjusted statistically for their impact on the relationship between exposure(s) and outcome(s)?
