# Supplementary material for: Exploring the Impact of Extracorporeal Membrane Oxygenation on the Endothelium: A Systematic Review
Source: Int J Mol Sci. 2024 Oct 3;25(19):10680. doi: 10.3390/ijms251910680 (PMC11477268; doi:10.3390/ijms251910680)
Supplement: Supplementary file 1 [file ijms-25-10680-s001.zip › Supplementary File S5.pdf]

Supplementary file S5. Changes of endothelial markers in patients with unfavorable outcomes vs. those without unfavorable outcomes

	markers	Study	Population	N	Type of ECMO	Day0	Day1	Day2	Day3	Day7	ND
Death	E-selectin	Caprarola et al.[20]	Pediatric	99	VA/VV		↓				~
	P-selectin	Caprarola et al.[20]	Pediatric	99	VA/VV		~				~
	VEGF	Tsai et al.[14]	Adult	23	VA	~	~	↓			
		Patry et al.[17]	Adult	16	VV						~
	Ang-2	Tsai et al.[14]	Adult	23	VA	↑	~	~			
		Patry et al.[17]	Adult	16	VV						↑
	Ang-1	Tsai et al.[14]	Adult	23	VA	~	~	~			
	Ang-2/Ang-1	Tsai et al.[14]	Adult	23	VA	~	~	~			
	Trombomodulin	Caprarola et al.[20]	Pediatric	99	VA/VV		~				~
		Tsai et al.[14]	Adult	23	VA	~	~	~			
	EVs	Siegel et al.[12]	Adult	18	VA		~				
Brain complications	E-selectin	Caprarola et al.[20]	Pediatric	99	VA/VV		~				~
	P-selectin	Caprarola et al.[20]	Pediatric	99	VA/VV		~				~

	VEGF	Xing et al.[19]	Pediatric	13	VV					↑	
	Ang-2/Ang-1	Xing et al.[19]	Pediatric	13	VV					~	
	sTie2	Xing et al.[19]	Pediatric	13	VV					↓	
	Trombomodulin	Caprarola et al.[20]	Pediatric	99	VA/VV		~				~
Hemorrhagic complications	P-selectin	Jang et al.[24]	Adult	132	VA/VV/VAV		~			↑	
	E-selectin	Jang et al.[24]	Adult	132	VA/VV/VAV		↑			↑	
	Trombomodulin	Jang et al.[24]	Adult	132	VA/VV/VAV		↑			↑	
Prolonged ECMO	ICAM-1	Pais et al.[22]	Pediatric	24	VA/VV	~	~	~	~		
	P-selectin	Pais et al.[22]	Pediatric	24	VA/VV	~	~	~	~		

↓ represents a significant decrease in ECMO patients with unfavorable outcomes (death, brain complications, hemorrhagic complications, prolonged ECMO support>7d) vs. those without. ↑ represents a significant increase in ECMO patients with unfavorable outcomes vs. those without. ~ represents no significant differences between patients with favorable and unfavorable outcomes.

ND not determined, *ECMO* extracorporeal membrane oxygenation, *VA* veno-arterial, *VV* veno-venous, *VAV* venoarterial-venous, *VEGF* vascular endothelial growth factor, *Ang-2* Angiopoietin 2, *Ang-1* Angiopoietin 1, *EVs* extracellular vesicles, *sTie2* soluble Tie2, *ICAM-1* intercellular adhesion molecule 1
